# High selectivity of photocatalytic reduction of CO_2_ to CO based on terpyridine ligand supported Cu^I^ metal organic framework

**DOI:** 10.3389/fchem.2022.974907

**Published:** 2022-08-05

**Authors:** Wen-Dong Zhang, Yun Wang, Yi Liang, Ai-Lin Jiang, Hao Gong, Xiao-Ying Tian, Wen-Sheng Fu, Jia-Zhen Liao, Peng Chen, Ying-Zhao Ma

**Affiliations:** ^1^ Chongqing Key Laboratory of Green Synthesis and Application, College of Chemistry, Chongqing Normal University, Chongqing, China; ^2^ Chongqing Key Laboratory of Inorganic Functional Materials, College of Chemistry, Chongqing Normal University, Chongqing, China; ^3^ Chongqing College of Electronic Engineering, Chongqing, China

**Keywords:** photocatalytic CO_2_ reduction, high selectivity, terpyridine ligand, Cu^I^ MOF, photocatalyst

## Abstract

In this work, a 4’-(4-cynaophenyl)-4,2’:6′,4-terpyridine supported Cu^I^ MOFs photocatalyst (**Cu** ^**I**^ **MOF**) was applied to the photocatalytic CO_2_ reduction for the first time. The micro-structural and physicochemical properties of the **Cu** ^**I**^ **MOF** were systematically studied by the powder X-ray diffraction (PXRD), Single crystal X-ray diffraction (SCXRD), scanning electron microscope (SEM), X-ray photoelectron spectroscopy (XPS), Fourier transform infrared (FT-IR), UV-Vis diffuse spectroscopy (UV-vis DRS), ns-level photoluminescence spectra (ns-level PL), Ultraviolet photoelectron spectroscopy (UPS), and N_2_ adsorption-desorption test (BET-BJH). Moreover, the *in situ* diffuse reflectance infrared fourier transform spectroscopy (*in situ* DRIFTS) was applied to investigate the adsorption and reaction intermediates of photocatalytic CO_2_ reduction. As a result, **Cu** ^**I**^ **MOF** exhibited good performance and outstanding selectivity toward photocatalytic reduction of CO_2_ to CO under full-spectrum and visible light illumination. Notably, 100% selective photocatalytic conversion of CO_2_ to CO was achieved. Thus, the study presents the high selectivity and CO_2_ reduction efficiency of **Cu** ^**I**^ **MOF** as a potential family of photocatalysts.

## Introduction

Photocatalytic CO_2_ reduction has been regarded as an especially promising approach in light of generating valuable chemical fuels to confront the waste gas CO_2_ issues (Chen et al., 2021; Jamjoum et al., 2021). Moreover, owing to its advantages such as the simplicity of utilization, good reusability, low cost, high efficiency and environmental friendliness, various photocatalysts have been designed and applied in photocatalytic CO_2_ reduction (Xuan et al., 2020). However, a large amount of photocatalysts still suffers from poor light absorption capacity, high recombination of photo-generated carriers and low selectivity of product, which limits their practical applications in photocatalytic CO_2_ reduction (Fu et al., 2019). Especially, selectivity of product plays the key role during photocatalytic CO_2_ reduction. As we all known, photocatalytic CO_2_ reduction involves a multi-electron reaction to generate a wide variety of products, including CO, CH_4_, CH_3_OH and HCOOH, etc., as well as even higher hydrocarbons (Li et al., 2019; Abbas and Sial, 2021) as carbon is in its highest oxidation state. Therefore, catalytic challenge is the precise tuning of the electron density to cater high selectivity. Attempts have been focused on the production of redox photosensitizers to transfer the excited electrons for the catalytic reduction of CO_2_. Although the first row transition metal, namely Fe, Co and Ni complexes (Cárdenas-Arenas et al., 2020; Zha et al., 2020; Liu et al., 2021), have been adopted to the substitution of low abundance metal analogues (such as Ru, Re, Os and Ir) (Deng et al., 2018; Deng et al., 2021; Karmakar et al., 2021) as multi-electron catalysts, their quick deactivation excited states (owing to the low lying d-d excited state) has limited the catalytic performance of the CO_2_ reduction. Very recently, the heteroleptic Cu^I^ coordination compounds has gained more attention due to their long lifetimes, showing strong metal-to-ligand (MLCT) excited state emission even in a solution at room temperature dominated by the Cu^I^ center’s d^10^ configuration nature (Yamazaki et al., 2019). Thus, the fine tuning of electron density around the metal center is of great significance. The photophysics and photochemistry of remote substituent effects in coordination compounds have been explored in the past by many groups (Fernández-Terán and Sévery, 2021a). Among them, Fernández-Terán and coworkers adopted 4‘-(4-substituted-phenyl)-terpyridine bearing substituents of different electrondonating abilities allowed the remote control of the electron density on the ligands. As the result, the readily tuning of ground- and excited-state properties of the resulting coordination compounds shows the potential of the terpyridine frameworks for high activity/selectivity of photocatalytic reduction chemistry (Fernández-Terán and Sévery, 2021b).

Meanwhile, metal-organic frameworks (MOFs) often possess high thermal and chemical stabilities and allow the incorporation of desired organic ligands featuring various electron-donating abilities through self-assembly and have been gradually applied in the field of catalysis (Zhang and Lin, 2014). Among the reported examples, Cu-based MOFs are highly concerned for its low-price and abundance in nature. In addition, Cu-MOF-based materials have gained extensive attentions as MOF-based catalysts for photocatalytic CO_2_ reduction. Wang et al. (Wang X.-K. et al., 2019). Reported that [Cu_3_(TCA)_2_ (dpe)_3_(H_2_O)_3_]_n_ material for photocatalytic CO_2_ reduction with the mixture products being CO and H_2_. Although the material exhibited good CO_2_ reduction activity (CO yield: 68.0 μmol g^−1^·h^−1^), the CO selectivity was merely 22.6%. He et al. (He and Wang, 2018). Fabricated Cu_3_(BTC)_2_-based photocatalysts which can efficiently reduce CO to a mixture of CO and CH_4_ (preferential product). Wang et al. (Wang L. et al., 2019). Synthesized PCN-224(Cu) for the photocatalytic reduction of CO_2_ under liquid-solid system, which possessed good light harvesting ability. The main reduction products of PCN-224(Cu) were CH_4_ and CO. However, although these Cu-MOF-based catalysts exhibited high CO_2_ reduction activity, the selectivity was poor. Therefore, it is highly desirable to further regulate Cu MOF catalysts to meet the high selectivity. While, similar to traditional heterogeneous and homogeneous catalysts, the reported Cu^I^-MOFs catalysts supported by non-terpyridine ligands also suffer from low CO_2_ photocatalytic activity/selectivity. This evokes us to study the photocatalytic CO_2_ reduction activity and selectivity of Cu^I^-MOFs supported by terpyridine ligands given the aforementioned high activity/selectivity potential of photocatalytic reduction. Thus, the 4’-(4-cynaophenyl)-4,2’:6′,4-terpyridine (**L**) supported Cu^I^ metal-organic-framework (**Cu** ^**I**^ **MOF**) reported by Hu and coworkers in 2005 is a good candidate in which **L** features a V-shaped large π-conjugated nonlinear structure. In addition, the cyano group in **L** could be substituted by different electron donating ability groups, allowing the remote control of the electron density to cater the high selectivity of multiple CO_2_ reduction products (Xi et al., 2015).

In this work, we adopted **Cu** ^**I**^ **MOF** as photocatalyst and its photocatalytic CO_2_ reduction activity and selectivity have been studied. It is notable that the **Cu** ^**I**^ **MOF** photocatalyst achieved 100% selcetive conversion of CO_2_ to CO. Furthermore, UV-vis DRS results indicated that **Cu** ^**I**^ **MOF** possesses good light absorption ability (200–800 nm). According to UPS and BET-BJH of **Cu** ^**I**^ **MOF**, suitable reduction potential position and high specific surface areas contributed to the high activity of the photocatalytic CO_2_ reduction. Finally, *in situ* DRIFT spectra revealed the possible mechanism of photocatalytic CO_2_ reduction of **Cu** ^**I**^ **MOF**. The synthesis of the **Cu** ^**I**^ **MOF** was modified and optimized to allow the delivery of smaller size of the crystalline material. This work not only demonstrated the outstanding photocatalytic CO_2_ reduction activity/selectivity of the terpyridine ligand supported Cu^I^ heteroleptic coordination compounds but also provide a promising strategy for potentially tuning of photocatalytic CO_2_ reduction selectivity product based on the ligand substituent group induced rich-electrondonating-diversity featuring terpyridine ligand supported Cu^I^-MOFs.

## Experimental section

### Materials

N,N-dimethylacetamide (DMA), ethanol, potassium hydroxide (KOH), ammonia solution and methanol were purchased from Chengdu kelong chemical Co., Ltd. Copper cyanide (CuCN), 4-acetylpyridine and 4-formylbenzonitrile were purchased from Shanghai macklin biochemical Co., Ltd. All reagents were directly used as received without further purification.

### Synthesis of L

Terpyridine ligand **L** was synthesized according to the literature method (Xi et al., 2015).

### Synthesis of Cu^I^ MOF

In a schlenk flask, the mixture of CuCN (1 mmol, 0.09 g), L (2 mmol, 0.70 g) and DMA (20 ml) was stirred under reflux for 12 h. Subsequently, the yellowish green suspension was allowed to cool down to room temperature and washed with DMA (2 × 20 ml), ethanol (2 × 20 ml) and deionized water (2 × 20 ml), in order, by centrifugation. Finally, the product was dried at 80 °C for 12 h (Yield: 0.54g, 67.9%). Anal. Calcd for C_108_H_63_Cu_9_N_27_ (%): C, 56.09; H, 2.73; N, 16.36. Found: C, 56.43; H, 2.89; N, 16.41.

### Activation of Cu^I^ MOF

The as-obtained Cu^I^ MOF sample needed be further activated (3 steps). Step 1: 0.30 g of Cu^I^ MOF was dispersed in 30 ml of DMA with 1 h stirring. Then, the suspension was transferred into a 50 ml Teflon-lined autoclave and heated to 80 °C for 24 h. Step 2: After the Cu^I^ MOF sample cooled down, the obtained catalyst was collected by centrifugation and activated again with ethanol. The activated steps were similar with Step 1, except that conditions was heated to 70 °C for 12 h. Step 3: After the sample cooled to room temperature, the obtained Cu^I^ MOF was collected by centrifugation and washed by ethanol (3 × 20 ml) and deionized water (3 × 20 ml). Next, the sample was dispersed with 5 ml of deionized water and frozen by liquid nitrogen. Finally, the sample was freeze-dried for 24 h.

### Characterization and analytical methods

The phase structure of sample was investigated by powder X-ray diffraction (PXRD: model D/max RA, Rigaku Co., Japan). Single crystal X-ray diffraction (SCXRD: Rigaku Oxford Diffraction, Rigaku Co., Japan) was applied to analyze single crystal structure. The morphology and micro-structure were studied with a scanning electron microscope (SEM: JEOL model JSM-6490, Japan). Fourier transform infrared (FT-IR) spectroscopy were implemented by the PerkinElmer Spectrum Two (U.K.), using KBr pellet and analyzing from 400 to 4,000 cm^−1^. The quantum efficiency and charge carrier lifetime of Cu^I^ MOF were conducted by a fluorescent spectrophotometer (FLS1000, Edinburgh Instruments, U.K.) with 450 nm of excited wavelength. The UV-Vis diffuse spectroscopy (UV-vis DRS: UV2550PC, Shimadzu, Japan) was used to characterize the optical properties. Ultraviolet photoelectron spectroscopy (UPS) measurements (Thermo Fisher Scientific) were carried out on valence band with using a He I (h*ν* = 21.2 eV) source. The surface chemical compositions and valence states were analyzed by X-ray photoelectron spectroscopy (XPS) measurements (K-alpha, Thermo Scientific). The specific surface area and pore volume of the sample were measured by the N_2_ adsorption-desorption specific surface analyzer (BET, BeiShiDe Instrument, BSD-PS). The elemental analysis for C, H, N were performed on a Perkin-elmer 240C analytical instrument.

### Photocatalytic CO_2_ reduction

The photocatalytic CO_2_ reduction experiment was evaluated using the Labsolar-6A system (Beijing Perfectlight). Before light irradiation, 10 mg of the catalyst was dispersed in 2 ml of pure water, and dropped by droplet onto a fiberglass filter and dried for using. After placing the dried sample in the reactor, the reactor was evacuated until no O_2_ or N_2_ could be detected via gas chromatography (kechuang, GC 2002). Then, the reactor filled with high-purity CO_2_ (≥99.999%, Chongqing lituoqiti Co., Ltd) several times and added 100 μL of deionized water, after which the reaction system pressure in the reactor was maintained at 90.0 ± 1.0 kPa. Then the reactor was exposed to the Xe lamp (lamp current: 20 A, PLS-SXE300+) full-spectrum light illumination for 8 h. The temperature of the entire reaction system was maintained at 20 ± 0.03°C through a recirculating cooling water system. The gaseous products were analyzed every 1 h on the gas chromatograph equipped with a methanizer, flame ionization detector (FID), and thermal conductivity detector (TCD), which could detect CO, CH_4_, and H_2_, O_2_, N_2_, respectively.

### *In situ* DRIFTS investigation on photocatalytic CO_2_ reduction

*In situ* DRIFTS measurements (diffuse reflectance infrared Fourier transform spectra) were conducted on a Nicolet iS50FT-IR spectrometer (Thermo Fisher, USA) equipped with a designed reactor and a liquid nitrogen cooled HgCdTe (MCT) detector. Then the loaded samples were purged with Ar (50 mlmin^−1^) for 1 h at 120°C to remove all the impurities. After the reactor temperature was dropped to room temperature, the background spectrum was collected. Next, the mixed gas (25 mlmin^−1^ of Ar, 5 mlmin^−1^ of CO_2_ and a trace of H_2_O vapor) was introduced into the reactor for about 30 min until reaching sorption equilibrium before illumination. Furthermore, the background spectrum was recorded again. Afterward, the turn on the light illumination (300 W Xe Lamp, AM 1.5 G) and the FTIR spectra were recorded as a function of time to investigate the dynamics of the conversion of the reactants under illumination.

## Results and discussion

### Phase structure

**FIGURE 1:**
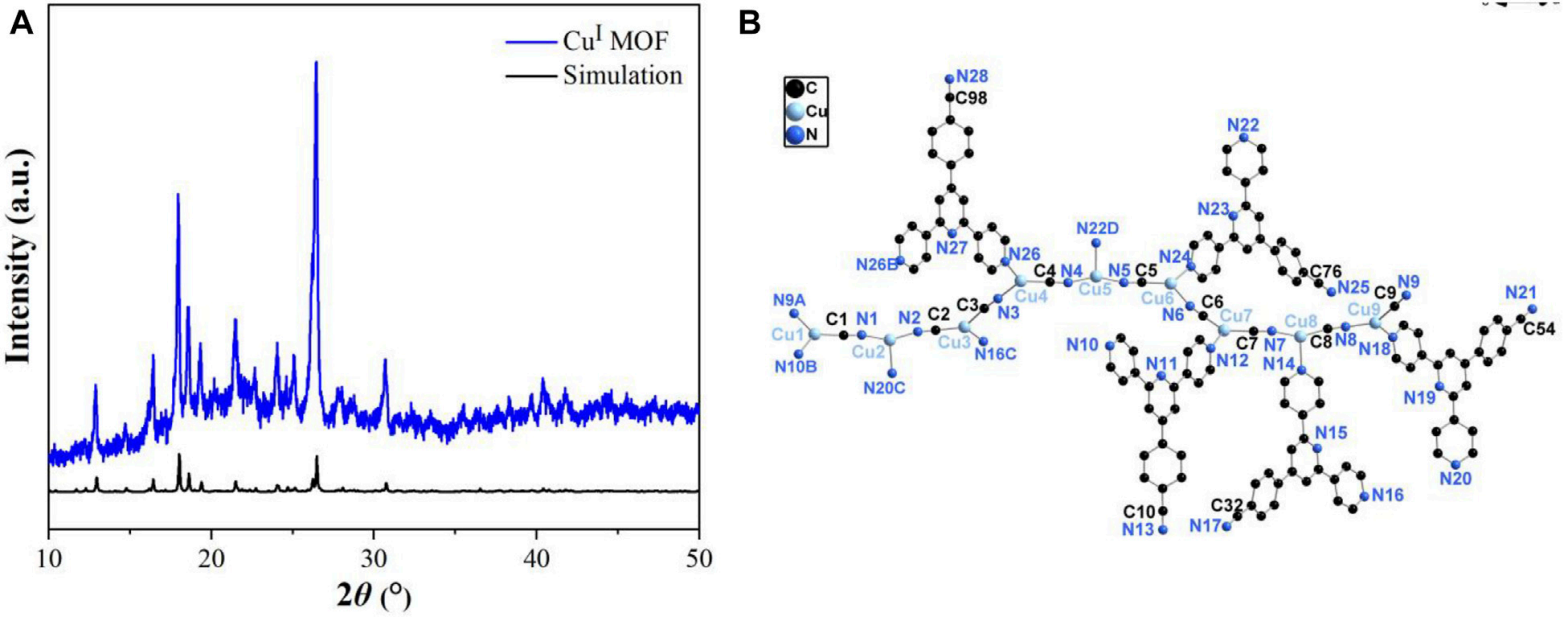
**(A)** Powder XRD pattern and **(B)** view of the coordination environments of **Cu** ^**I**^ **MOF**.

As shown in Figure 1A, the characteristic diffraction peaks can be well matched with single crystal simulation (SIM) patterns, which not only indicating the successful synthesis of **Cu** ^**I**^ **MOF**, but also conformed the phase purity of it. Notably, the considerably sharp diffraction peaks of **Cu** ^**I**^ **MOF** reveals the good crystallinity. The structure illustration of **Cu** ^**I**^ **MOF** was presented in Supplementary Figure S1 and Figure 1B. **Cu** ^**I**^ **MOF** is a three dimensional interpretation structure. The asymmetric unit comprised by nine Cu^I^ ions, four and a half ligands and nine cyanide ions with all the bond lengths and angles are identical to the structure reported (Xi et al., 2015).

### Micro-structure and morphology

**FIGURE 2:**
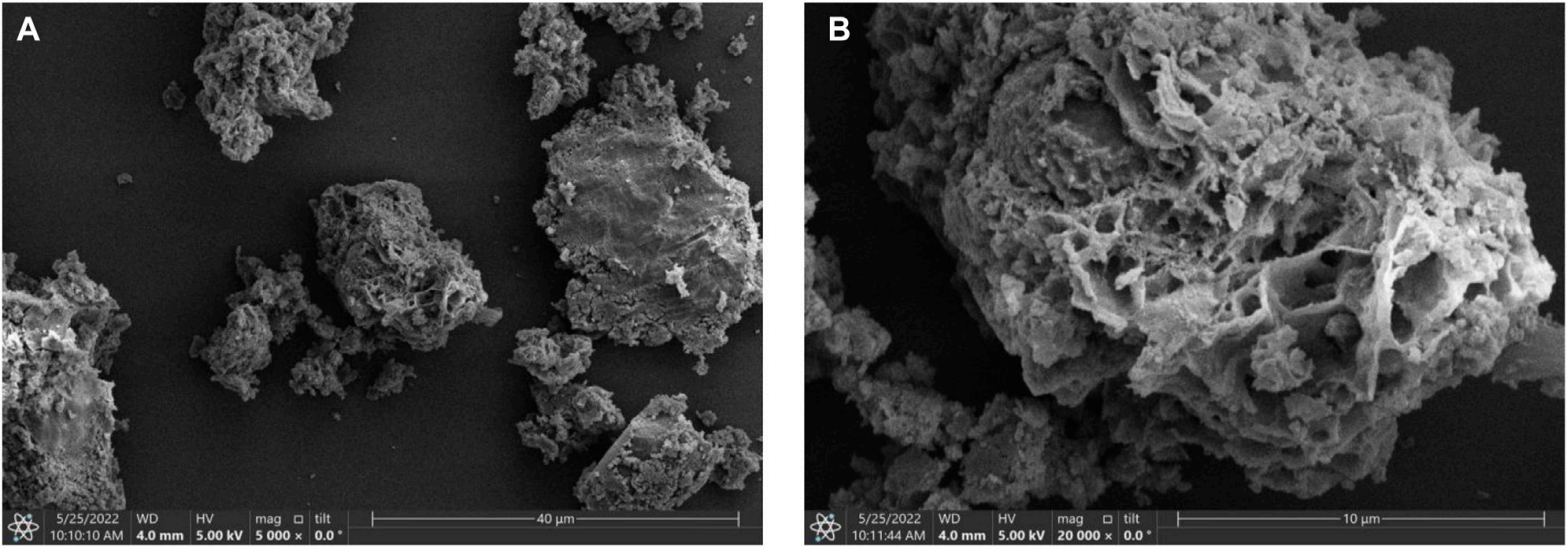
SEM images of **Cu** ^**I**^ **MOF**. Scale bar: 40 µm for **(A)**; 10 µm for **(B)**.

SEM was carried out to explore the micro-structures and morphologies of the **Cu** ^**I**^ **MOF**. As depicted in Figure 2A, the as-obtained **Cu** ^**I**^ **MOF** demonstrates irregular and fluffy porous structure. Figure 2B further confirms that the tremella-like morphology of **Cu** ^**I**^ **MOF** is self-assembled by plenty of the stacked nanosheets and nanoparticles.

### FT-IR spectra

**FIGURE 3:**
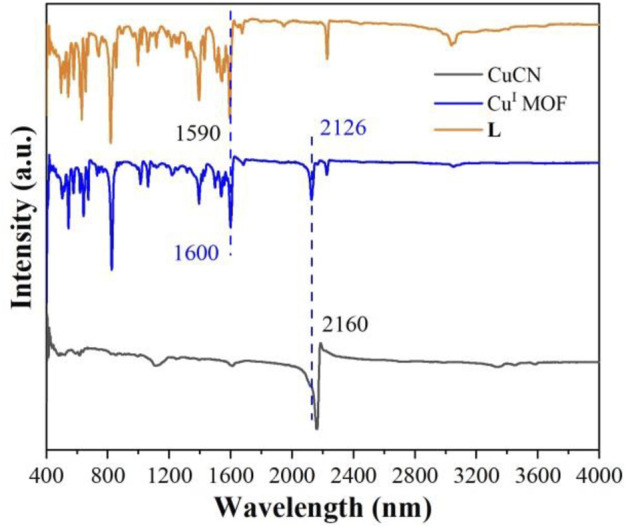
FT-IR spectra of **Cu** ^**I**^ **MOF.**

The FT-IR spectra of the as-prepared **Cu** ^**I**^ **MOF**, CuCN and **L** were illustrated in Figure 3. The significant peaks at 1,590 and 1,600 cm^−1^ were attributed to the C=C/C=N stretching vibration of pyridine (Singh et al., 2021). The band at 2,126 and 2,160 cm^−1^ were ascribed to the C≡N stretching vibration (Zhang et al., 2018). Compared to CuCN (2,160 cm^−1^) and **L** (1,590 cm^−1^), the C≡N and C=C/C=N characteristic peaks of **Cu** ^**I**^ **MOF** shifted, respectively, suggesting that CuCN and **L** are coodinated. Moreover, the existence of CuCN and **L** characteristic peaks of **Cu** ^**I**^ **MOF** sample indicated that **Cu** ^**I**^ **MOF** was successfully synthesized by this method.

### XPS

**FIGURE 4:**
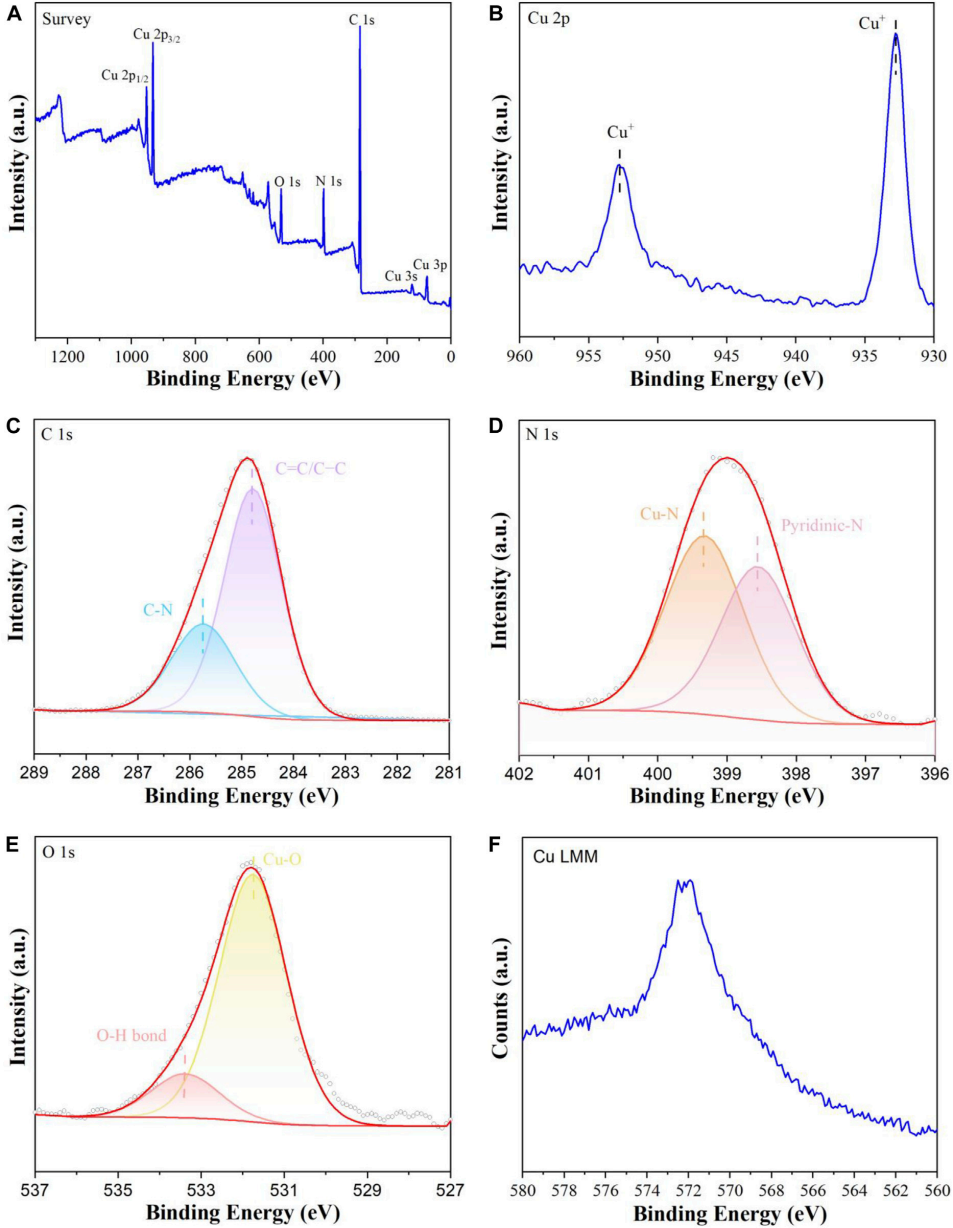
**(A)** XPS survey spectra of **Cu** ^**I**^ **MOF**, high resolution spectra of **(B)** Cu 3d, **(C)** C 1s, **(D)** N 1s, **(E)** O 1s, **(F)** Cu LMM Auger spectrum for in **Cu** ^**I**^ **MOF**.

The XPS was carried out to investigate the surface chemical compositions and valence states of **Cu** ^**I**^ **MOF** sample. As can be seen from Figure 4A, the XPS survey spectrum reveals the presence of C, N, O and Cu elements in **Cu** ^**I**^ **MOF** sample. In the high resolution Cu 2p spectra (Figure 4B), the main peaks at 952.6 and 932.8 eV are assigned to Cu^I^ (Zhu et al., 2021). To further identify Cu^I^, Cu LMM Auger spectrum as shown in Figure 4F. The Cu Auger LMM peak was observed at 571.9 eV (Jiang et al., 2022) in the binding energy scale, which was corresponded to the characteristic peak of Cu^I^. These results show that the valance of copper existed as Cu^I^ in **Cu** ^**I**^ **MOF.** As displayed in Figure 4C, the peaks at 285.7 and 284.8 eV in the high resolution C 1s spectra are ascribed to the C-N and C=C/C-C bond in the **L** (Zhu et al., 2021). The high resolution N 1s spectra of **Cu** ^**I**^ **MOF** sample is provided in Figure 4D. Binding energy at 399.3 and 398.6 eV are indexed to Cu-N bond (Zhu et al., 2021) and pyridinic N (Singh et al., 2021), respectively. The formation of Cu-N bond proved that the Cu elements were successfully coordinated with the cyano groups and the N in the **L**. The high resolution O 1s spectra is demonstrated in Figure 4E. It can be found that the binding energies at 533.4 and 531.8 eV correspond to O-H bonds of surface absorption water and Cu-O (H_2_O) interactions (Liu et al., 2021), respectively.

### UV-vis DRS and ns-level PL

**FIGURE 5:**
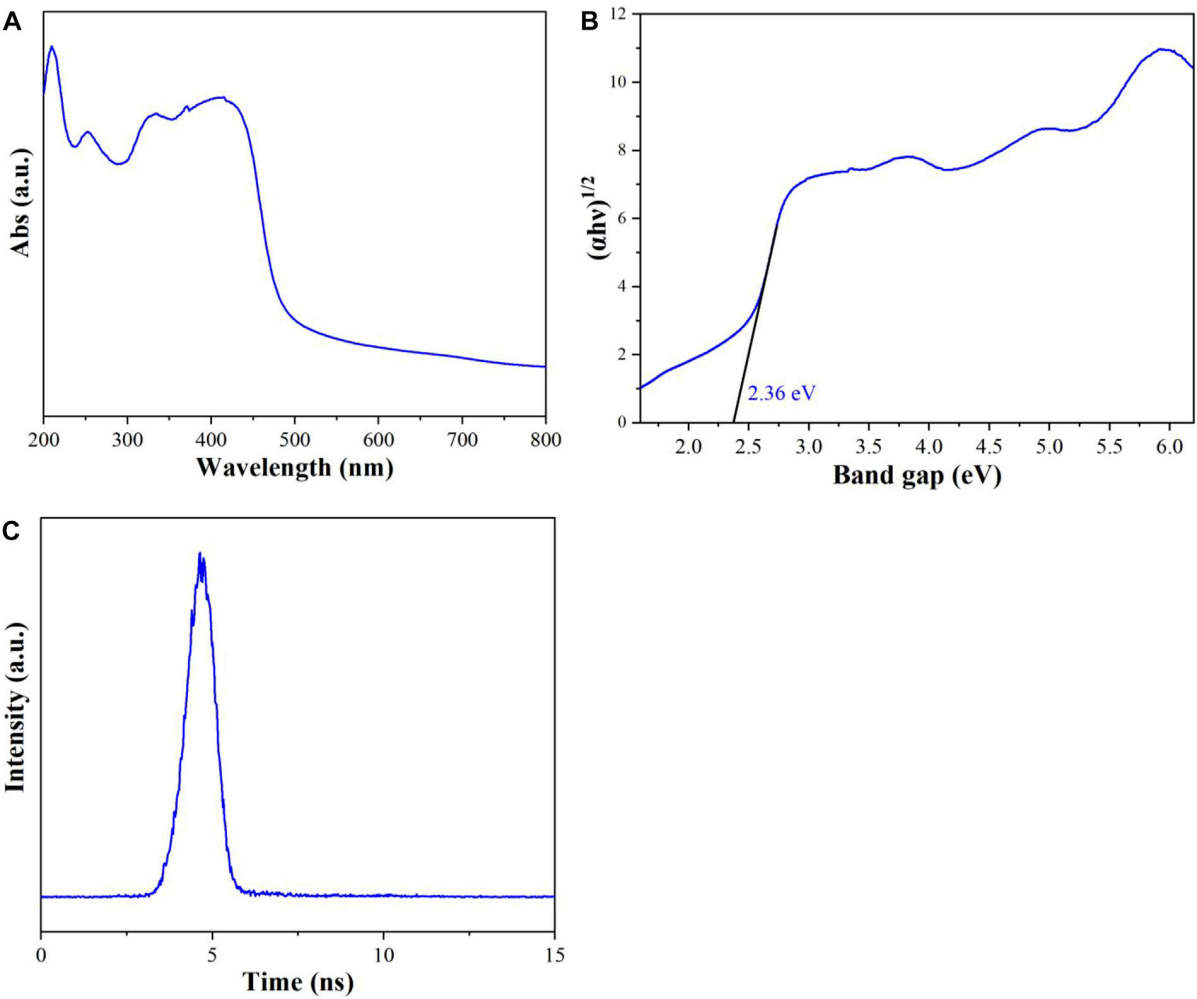
**(A)** UV-vis diffuse reflectance spectra, **(B)** plots of (αhν)^1/2^ νs. Photo energy and **(C)** time-resolved PL spectra monitored at under 450 nm excitation at 298 K for **Cu** ^**I**^ **MOF.**

The UV-vis diffuse reflectance spectra of **Cu** ^**I**^ **MOF** have been conducted to evaluate its light absorption ability (Figure 5A). **Cu** ^**I**^ **MOF** displayed good absorption ability in the ultraviolet and visible region, which is in good consistent with the color of **Cu** ^**I**^ **MOF** sample (inset of Figure 5A). In Figure 5B, the band gap of **Cu** ^**I**^ **MOF** has been calculated from the intercept of the tangents to the plots of (αhν)^1/2^ νs. Photo energy is 2.36 eV. The time-resolved PL spectra as depicted in the Figure 5C, the carrier lifetime of **Cu** ^**I**^ **MOF** is 0.08 ns? Furthermore, the quantum efficiency of **Cu** ^**I**^ **MOF** is 1.32% under visible light (450 nm) illumination.

### UPS

**FIGURE 6:**
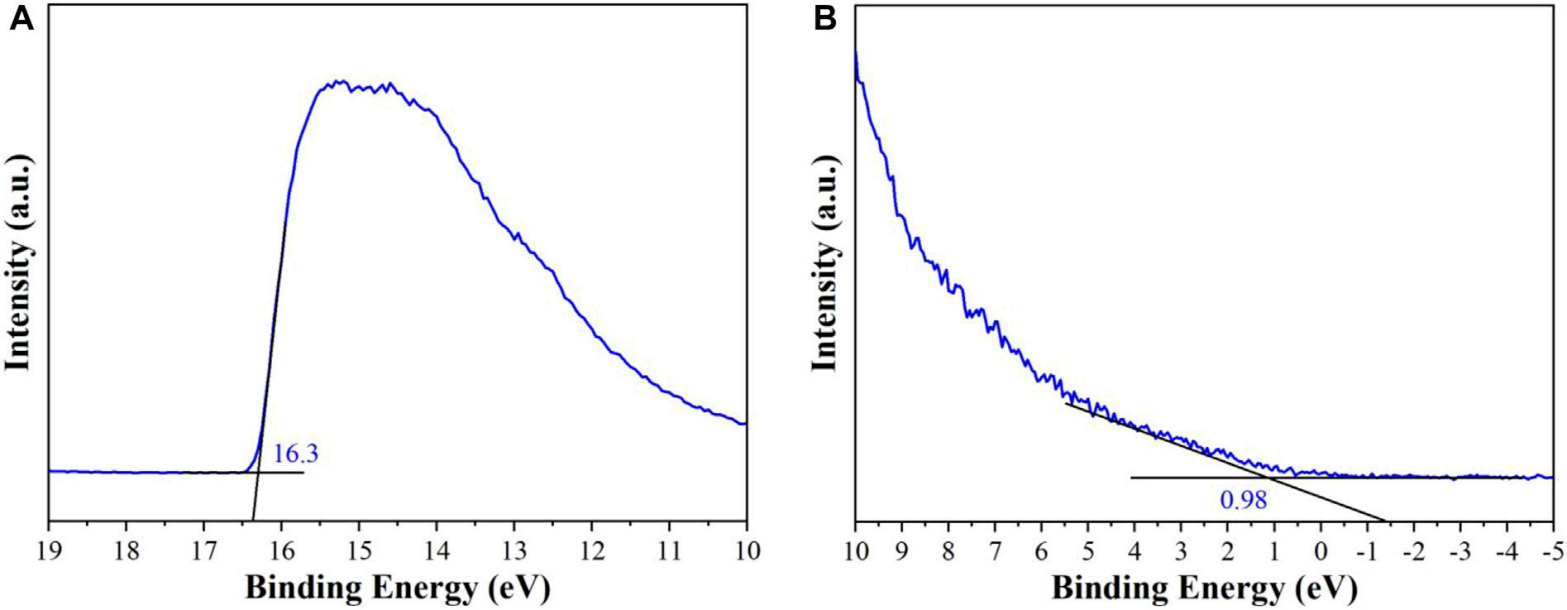
**(A)** The secondary edge region and **(B)** the HOMO region of UPS spectra of **Cu** ^**I**^ **MOF**.

UPS was conducted to investigate the position of valence band (VB) and conduction band (CB). The abscissa is the binding energy which is relative to the Fermi energy (E_F_) of Au. It is defined by the energy of the electron before excitation relative to the vacuum level. The high binding energy cutoff (E_cutoff_) of **Cu** ^**I**^ **MOF** is illustrated in Figure 6A. E_cutoff_ is decided by linear extrapolation to zero of the yield of secondary electrons. In Figure 6A, E_cutoff_ = 16.3 ± 0.03 eV for **Cu** ^**I**^ **MOF**. The HOMO region for **Cu** ^**I**^ **MOF** is observed in Figure 6B. The HOMO energy is decided using the incident photon energy, h*ν* = 21.2 eV, E_cutoff_, and the E_onset_ (the onset of **Cu** ^**I**^ **MOF** relative to the E_F_ of Au). In Figure 6B, E_onset_ = 0.98 ± 0.03 eV for **Cu** ^**I**^ **MOF**. The HOMO energy is thus gained directly from the UPS measurement, E_HOMO_ = h*ν*-(E_cutoff_ - E_onset_) (Gong et al., 2011; Sun et al., 2011; Zheng et al., 2018).

For **Cu** ^**I**^ **MOF**, E_HOMO_ = -5.88 ± 0.06 eV. The LUMO energy is calculated using the HOMO levels and the optical gaps (*E*_g_) obtained from UV-Vis DRS (Figure 5B). Thus, the E_LUMO_ = -3.52 ± 0.06 eV is for **Cu** ^**I**^ **MOF**. Concequnently, the photo-generated electrons of **Cu** ^**I**^ **MOF** could reduce CO_2_ to CO. According to the characterization results of UPS and UV-vis diffuse reflectance spectra (Figure 5A, Figure 6), the diagram of band structure is shown in Figure S4.

### BET-BJH

**FIGURE 7:**
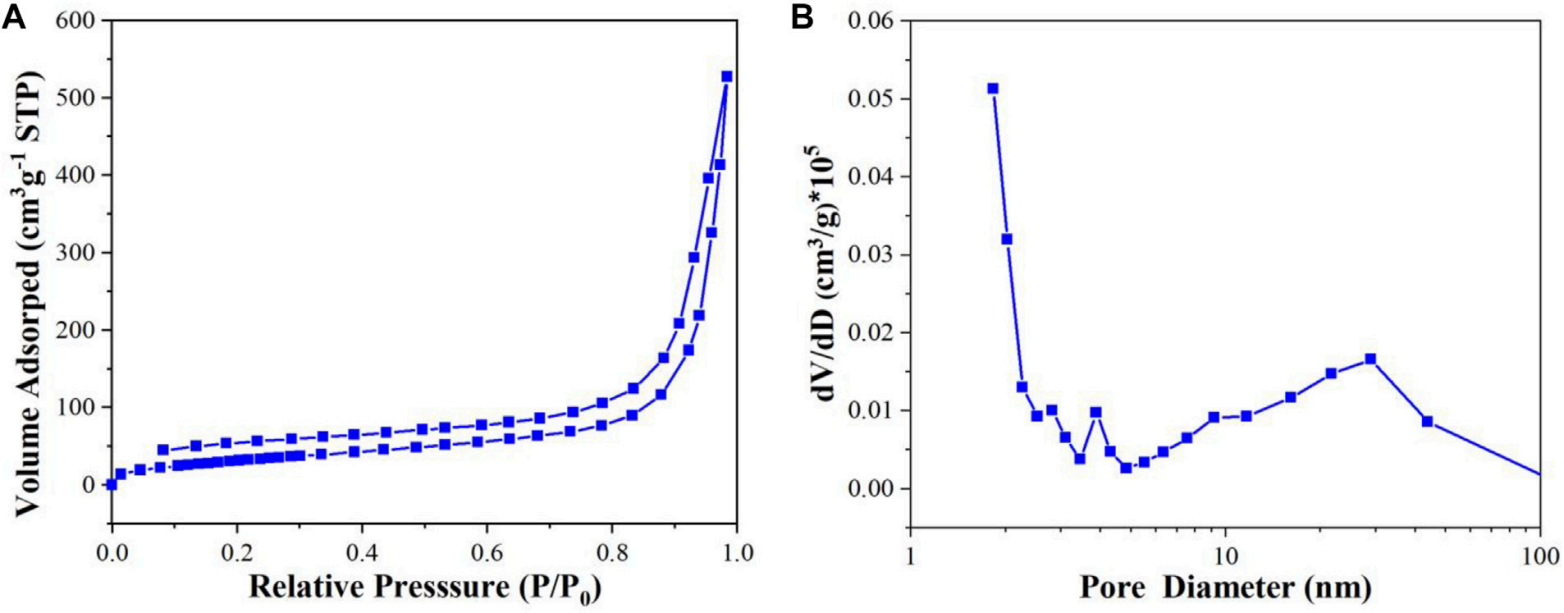
**(A)** N_2_ adsorption-desorption isotherm curves and **(B)** pore size distributions of **Cu** ^**I**^ **MOF**.

As can be seen from Figure 7A, the N_2_ adsorption-desorption isotherm of **Cu** ^**I**^ **MOF** exhibits typical IV isotherms with H3 type hysteresis loop, which indicates the existence of mesopores. The pore size distribution curve further demonstrates the existence of mesopores (Figure 7B). The specific surface area, corresponding pore volume and average pore diameter are 116.98 m^2^ g^−1^, 0.77 cm^3^ g^−1^ and 26.40 nm respectively. The high specific surface area and large pore volume could provide more active sites for the reactant adsorption and photocatalytic reaction, which is in agreement with SEM result.

### Photocatalytic performance

**FIGURE 8:**
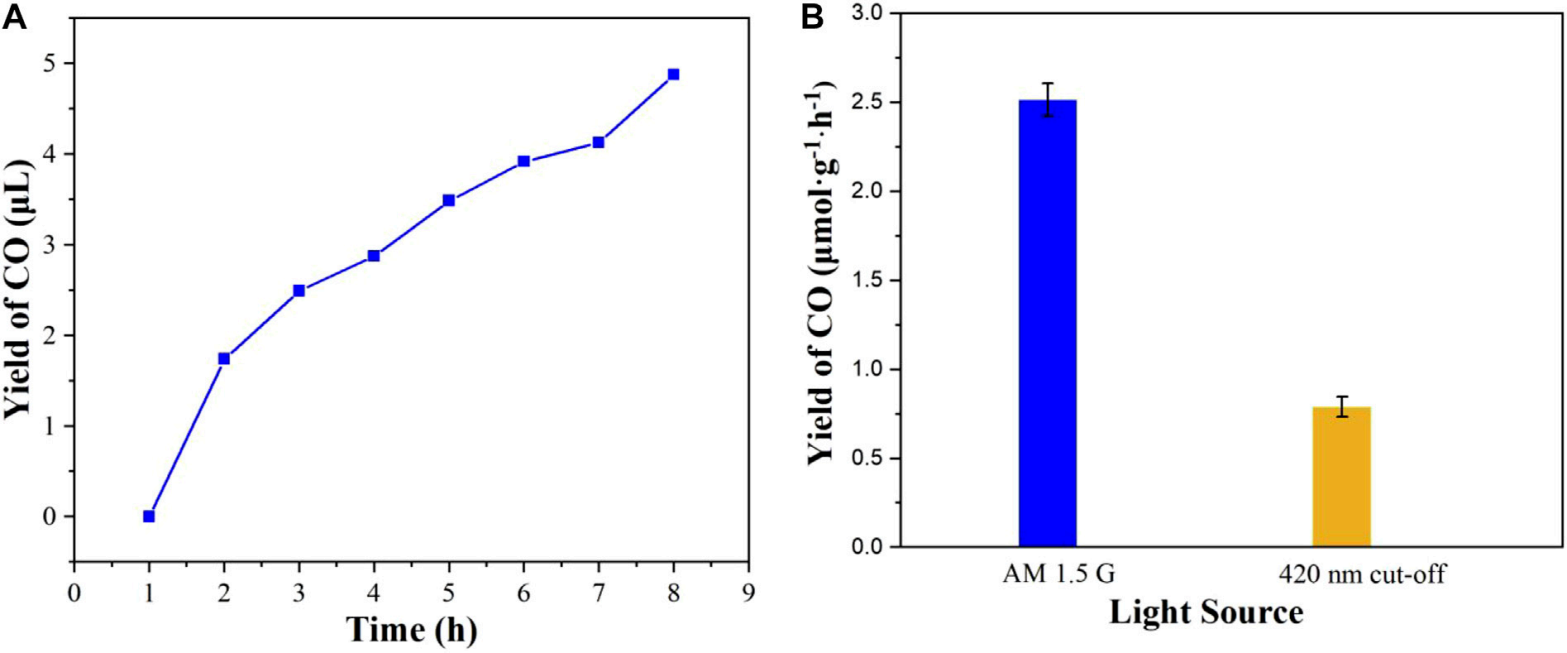
**(A)** The yields of CO reduced by CO_2_ under AM 1.5 G illumination and **(B)** the yields rate of CO under different light source illumination.

The experiment of full-spectrum light (AM 1.5 G) driven CO_2_ reduction was performed to evaluate the photocatalytic activity. Before the beginning of photocatalytic CO_2_ reduction, no CO and other organic matter are detected under the reaction conditions of without light, photocatalyst and with Ar atomosphere, respectively, demonstrating that CO_2_ is the sole carbon source for the reaction. As shown in Figure 8A, it is interesting that the CO concentration was found to increase gradually with the extension of the illumination time. After 8 h of photocatalytic reaction, no other product can be detected additional to CO, which suggests that the selectivity of CO production is 100%. It is worthwhile mentioning that the yield rate of CO is 2.58 μmol g^−1^·h^−1^ under 8 h full-spectrum light illumination. Remarkably, **Cu** ^**I**^ **MOF** also shows a good visible light photocatalytic activity for CO_2_ reduction, whose yield rate of CO is 1.83 μmol g^−1^·h^−1^ under 8 h illumination (Figure 8B). Moreover, the photocatalytic stability of **Cu** ^**I**^ **MOF** was assessed through three sequent tests of photocatalytic CO_2_ reduction for 24 h (Supplementary Figure S2). The yield of CO dropped from about 2.58 to 0.87 μmol g^−1^·h^−1^, which suggested poor photocatalytic stability. The poor photocatalytic stability could be due to photo-destabilization of functional groups (Feng et al., 2020), which decomposed the framework of **Cu** ^**I**^ **MOF** and caused poor photocatalytic stability.

### Mechanisms of photocatalytic CO_2_ reduction

**FIGURE 9:**
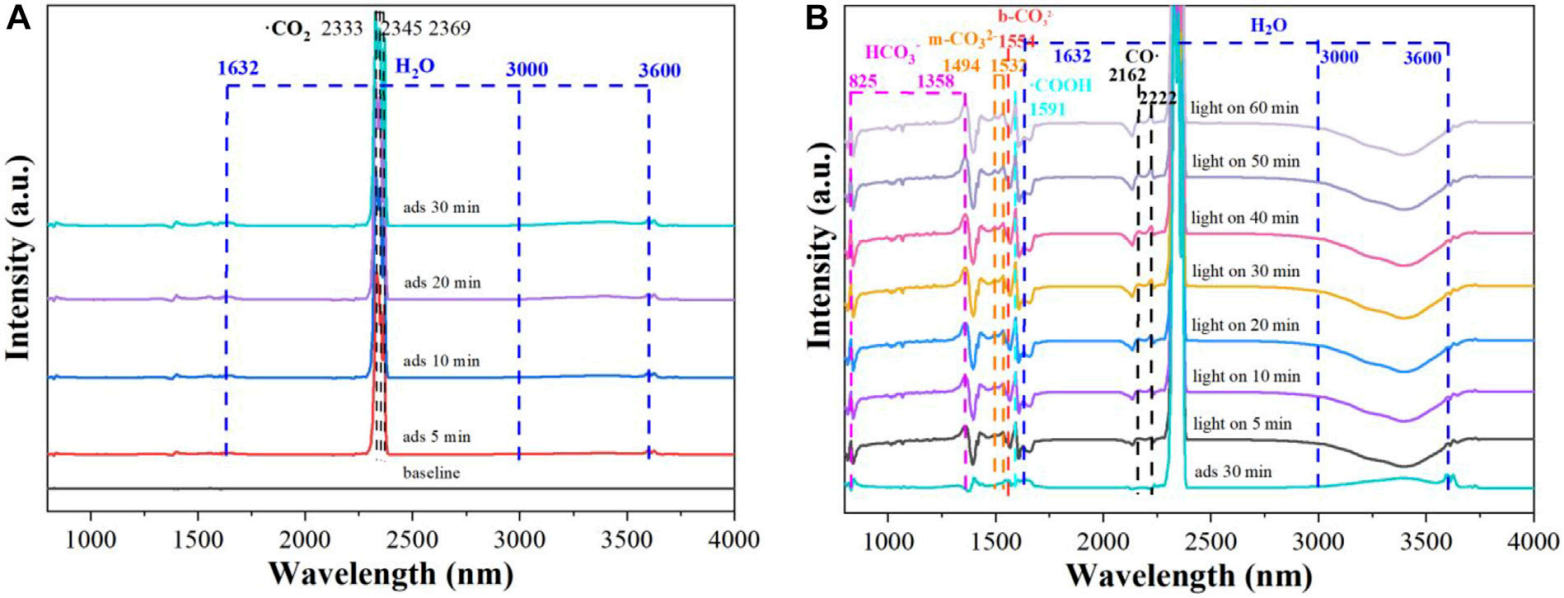
**(A)** *In situ* DRIFT spectra of CO_2_ and H_2_O absorption and **(B)** reaction on **Cu** ^**I**^ **MOF**.

As can be seen in Figure 9A, the peaks at 2,333, 2,345 and 2,369 cm^−1^ are indexed to CO_2_ (Coenen et al., 2018) and the peaks at 1,632 and 3,000–3,600 cm^−1^ are attributed to H_2_O (Tang et al., 2019; Zhao Y. et al., 2020), respectively. Furthermore, the intensities of the peaks increase with prolonged absorption time, which indicates that CO_2_ molecules are activated on the surface of **Cu** ^**I**^ **MOF**.

In Figure 9B, after light turned on, a number of intermediates are detected, including HCO_3_^−^ (825 and 1,358 cm^−1^) (Liu et al., 2017; Ma et al., 2017), b-CO_3_^2−^ (1,554 cm^−1^) (Tang et al., 2019), m-CO_3_^2−^ (1,494 and 1,532 cm^−1^) (Liu et al., 2017), −COOH (1,591 cm^−1^) (Zhao J. et al., 2020) and CO (2,162 and 2,222 cm^−1^) (Ma et al., 2017). Moreover, the intensities of thoes peaks gradually enhanced with the extension of illumination time. Accordingly, the possible CO_2_ conversion pathway was proposed as following:

$$CO_{2}+H_{2}O\to \mathrm{HC}O_{3}^{–}+H^{+}$$ (1)

$${\text{HCO}}_{3}^{\text{–}}\to {\text{CO}}_{3}^{2-}{+\text{H}}^{+}$$ (2)

$${\text{CO}}_{2}↑\to •{\text{CO}}_{2}$$ (3)

$$•CO_{2}+H^{+}+e^{-}\to •\mathrm{COOH}$$ (4)

$$•{\text{COOH}+\text{H}}^{+}{+\text{e}}^{-}\to \text{CO}•$$ (5)

CO•→CO↑ (6)

## Conclusion

In summary, the synthesis of **Cu** ^**I**^ **MOF** was successfully optimized for photocatalytic CO_2_ reduction. The CO evolution reached 2.58 μmol g^−1^·h^−1^ and achieved 100% conversion. Moreover, UV-vis DRS result indicates that **Cu** ^**I**^ **MOF** displays good light absorption capacity (200–800 nm). The BET-BJH result reveals that **Cu** ^**I**^ **MOF** possesses high specific surface area (116.98 m^2^ g^−1^) and large pore volume (0.77 cm^3^ g^−1^). The position of conduction band (-3.52 ± 0.06 eV) of **Cu** ^**I**^ **MOF** is negative enough to meet the photocatalytic CO_2_ reduction. Finally, the possible mechanisms of photocatalytic CO_2_ reduction were revealed by the *in situ* DRIFTS. This study demonstrated the promising potential of terpyridine ligand supported Cu^I^-MOFs for the high activity/selectivity of photocatalytic CO_2_ reduction.

## Data Availability

The original contributions presented in the study are included in the article/Supplementary Material; further inquiries can be directed to the corresponding authors.
