# Supplementary material for: High selectivity of photocatalytic reduction of CO2 to CO based on terpyridine ligand supported CuI metal organic framework
Source: Front Chem. 2022 Aug 5;10:974907. doi: 10.3389/fchem.2022.974907 (PMC9388720; doi:10.3389/fchem.2022.974907)
Supplement: Supplementary file 1 [file DataSheet1.PDF]

Supplementary Material

High Selectivity of Photocatalytic Reduction of CO_2 to CO based on Terpyridine Ligand Supported Cu^{I} Metal Organic Framework

Wen-Dong Zhang[1], Yun Wang[2†], Yi Liang[2†], Ai-Lin Jiang[2†], Hao Gong[1], Xiao-Ying Tian[1], Yi Wang[1], Wen-Sheng Fu[1*], Jia-Zhen Liao[3], Peng Chen[2*], Ying-Zhao Ma[1*]

[1] Chongqing Key Laboratory of Green Synthesis and Application, College of Chemistry, Chongqing Normal University, Chongqing, China

[2] Chongqing Key Laboratory of Inorganic Functional Materials, College of Chemistry, Chongqing Normal University, Chongqing, China

[3] Chongqing College of Electronic Engineering, Chongqing, China

[4] Research Center for Environmental Science & Technology, Institute of Fundamental and Frontier Sciences, University of Electronic Science and Technology of China, Chengdu, China

*** Correspondence:**
Ying-Zhao Ma
yzma@cqnu.edu.cn
Wen-Sheng Fu
fuwensheng@cqnu.edu.cn
Peng Chen
ctbupengchen@126.com

[†]These authors have contributed equally to this work and share first authorship

1 Crystallographic appendix

Crystallographic data sets were collected with either Mo K_α radiation (0.71073 Å) .Suitable single crystals were covered in parabar oil, placed on a nylon loop mounted in a CrystalCap MagneticTM holder (Hampton Research) and immediately transferred to the diffractometer. Data were corrected for Lorentz and polarization effects, as well as for absorption (numerical integration using a Gaussian grid, including beam profile effects). The program suite CrysAlisPRO (Rigaku Oxford Diffraction) was used for data collection, multi-scan absorption correction and data reduction. Each structure was solved by intrinsic phasing using SHELXT-2016 (Sheldrick, 2015b) and refined by full-matrix least-squares methods on F^2 with SHELXL2016 (Sheldrick, 2015a) in the software OLEX2. The structures were checked for higher symmetry with the help of the program PLATON (Spek, 2003). After anisotropic refinement of the non-H atoms, hydrogens were placed in geometrically calculated positions and each was assigned a fixed isotropic displacement parameter with a value equal to 1.2Uiso of its parent C atom (1.5U_{iso} for methyl groups) and refined with a riding model.

Table S1. Crystallographic table

Compound	**Cu^{I} MOF**
Formula	$C_{108}H_{63}Cu_{9}N_{27}$
μ/mm^{-1}	2.683
Formula weight	2310.81
Color	Yellow
Shape	Block
Size/mm^{3}	0.04 × 0.03 × 0.01
T/K	100(2)
Crystal system	orthorhombic
Space group	Pbcn
a/Å	20.6917(6)
b/Å	14.3705(3)
c/Å	64.916(2)
α/°	90
β/°	90
γ/°	90
Volume/Å^{3}	19302.9(9)
Z	2
Wavelength/Å	1.54184

Radiation type	Cu Kα (λ = 1.54184)
Θ_{min}/°	5.064
Θ_{max}/°	140.432
Measured Refl.	62989
Independent Refl.	16600
Reflections Used	18248
R_{int}	0.01221
Parameters	1300
Restraints	72
Largest Peak	1.29
Deepest Hole	-1.14
GooF	1.438
wR_2 (all data)	0.3927
wR_2	0.3265
R_1 (all data)	0.1647
R_1	0.1158
ρ_{calc}g/cm^3	1.590
μ/mm^{-1}	2.638
F(000)	9288.0

Radiation	Cu Kα (λ = 1.54184)
2Θ range for data collection/°	5.064 to 140.432
Index ranges	$-21 \leq h \leq 24$, $-16 \leq k \leq 16$, $-76 \leq l \leq 74$
Reflections collected	62989
Independent reflections	16600 [R_{int} = 0.1221, R_{sigma} = 0.0678]
Data/restraints/parameters	16600/72/1300
Goodness-of-fit on F^2	1.438
Final R indexes [I>=2σ (I)]	R_1 = 0.1158, wR_2 = 0.3265
Final R indexes [all data]	R_1 = 0.1647, wR_2 = 0.3927
Largest diff. peak/hole / e $Å^{-3}$	1.29/-1.14

Table S2. Selected Bond Distances (Å) and angles (°) for Cu^{I} MOF

**Cu^{I} MOF**					
Cu1-N9^{2}	1.949(11)	Cu4-N3	1.951(12)	Cu6-N24	2.018(7)
Cu1-N10^{1}	2.029(8)	Cu4-N26	2.030(8)	Cu7-N12	2.047(9)
Cu2-N1	1.881(8)	Cu5-N4	1.877(8)	Cu8-N7	1.892(9)
Cu2-N2	1.890(8)	Cu5-N5	1.885(7)	Cu8-N14	2.114(7)
Cu2-N20^{3}	2.095(7)	Cu5-N22^{4}	2.109(7)	Cu9-N8	1.896(10)
Cu3-N16^{3}	2.035(7)	Cu6-N6	1.968(10)	Cu9-N18	2.030(7

C1-Cu1-N92	127.3(3)	N5-Cu5-N224	102.2(3)
C1-Cu1-N101	125.7(3)	C5-Cu6-N6	125.6(3)
N92-Cu1-N101	107.0(3)	C5-Cu6-N24	133.8(3)
N1-Cu2-N2	154.3(3)	N6-Cu6-N24	100.3(3)
N1-Cu2-N203	103.6(3)	C6-Cu7-C7	134.2(4)
N2-Cu2-N203	102.0(3)	C6-Cu7-N12	110.2(4)
C2-Cu3-C3	131.3(4)	C7-Cu7-N12	115.6(4)
C2-Cu3-N163	127.8(3)	C8-Cu8-N7	153.9(4)
C3-Cu3-N163	100.7(3)	C8-Cu8-N14	104.0(3)
C4-Cu4-N3	129.4(4)	N7-Cu8-N14	102.1(3)
C4-Cu4-N26	127.4(4)	C9-Cu9-N18	106.5(3)
N3-Cu4-N26	103.1(3)	N8-Cu9-C9	131.2(4)

N4-Cu5-N5	156.5(3)	N8-Cu9-N18	122.3(3)
N4-Cu5-N224	101.2(3)		

Symmetry code for **1**: [1]1-X,+Y,3/2-Z; [2]-1+X,1-Y,1/2+Z; [3]1-X,-Y,1-Z; [4]1-X,2-Y,1-Z;

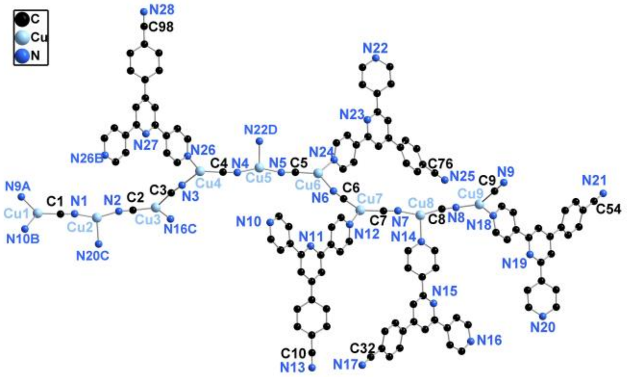

Figure S1. View of the coordination environments of compound 1. All H atoms and lattice water molecules are omitted for clarity. Symmetry codes: A (-1+x, 1-y, 1/2+z); B (1-x, y, 3/2-z); C (1-x, -y, 1-z); D (1-x, 2-y, 1-z)

2 Photocatalytic stability of Cu^{I} MOF

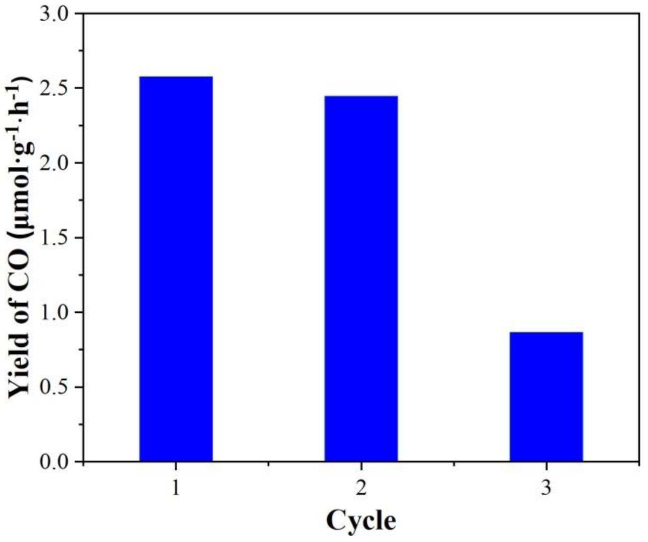

Figure S2. The cycling activity test of **Cu^{I} MOF**

3 The band structure of Cu^{I} MOF

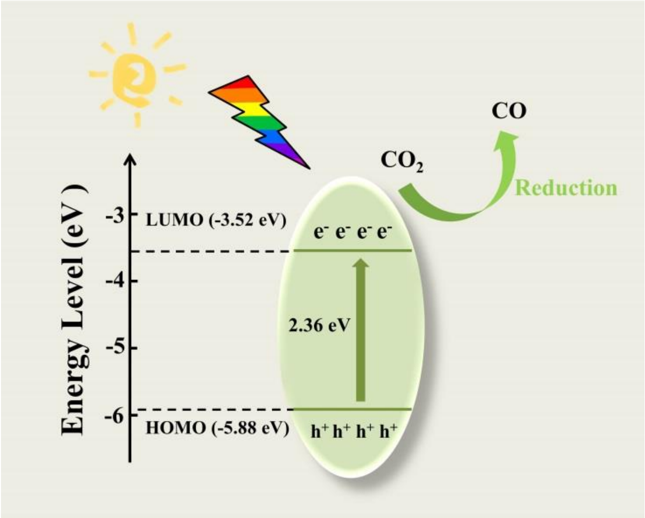

Figure S3. The diagram of band structure for **Cu^{I} MOF**

References

SHELDRICK, G. 2015a. Crystal structure refinement with SHELXL. *Acta Crystallographica Section C*, 71**,** 3-8.

SHELDRICK, G. 2015b. SHELXT - Integrated space-group and crystal-structure determination. *Acta Crystallographica Section A*, 71**,** 3-8.

SPEK, A. 2003. Single-crystal structure validation with the program PLATON. *Journal of Applied Crystallography,* 36**,** 7-13.
